# Prevalence and Sociodemographic Variation of Allergic Diseases in Australia: Findings From the Australian National Health Survey

**DOI:** 10.1111/cea.70288

**Published:** 2026-04-05

**Authors:** Yichao Wang, Jennifer J. Koplin, Janet M. Davies, Constance H. Katelaris, Debra J. Palmer, Jason A. Trubiano, Joy Lee, Michaela Lucas, Michael O′Sullivan, Sandra Vale, Sheryl van Nunen, Troy Wanandy, Dianne E. Campbell, Kirsten P. Perrett, Rachel L. Peters

**Affiliations:** ^1^ Deakin Lifespan Institute, School of Psychology Deakin University Geelong Victoria Australia; ^2^ Department of Paediatrics University of Melbourne Parkville Victoria Australia; ^3^ Population Allergy, Murdoch Children's Research Institute Parkville Victoria Australia; ^4^ National Allergy Centre of Excellence (NACE) Murdoch Children's Research Institute Parkville Victoria Australia; ^5^ Child Health Research Centre, University of Queensland Brisbane Queensland Australia; ^6^ Centre for Immunology and Infection Control, Queensland University of Technology Queensland Australia; ^7^ School of Medicine, Western Sydney University Sydney New South Wales Australia; ^8^ Immunology Unit, Department of Medicine Campbelltown Hospital Sydney New South Wales Australia; ^9^ The Kids Research Institute Australia, University of Western Australia Nedlands WA Australia; ^10^ School of Medicine, University of Western Australia Crawley WA Australia; ^11^ Centre for Antibiotic Allergy and Research, Department of Infectious Diseases and Immunology, Austin Health Heidelberg Victoria Australia; ^12^ Allergy, Asthma and Clinical Immunology, Alfred Health Melbourne Victoria Australia; ^13^ School of Translational Medicine, Monash University Melbourne Victoria Australia; ^14^ Immunology Department, Perth Children's Hospital Perth WA Australia; ^15^ Medical School, University of Western Australia Perth WA Australia; ^16^ Department of Immunology PathWest Laboratory Medicine Perth WA Australia; ^17^ Department of Immunology Sir Charles Gardiner Hospital Perth WA Australia; ^18^ National Allergy Council Sydney New South Wales Australia; ^19^ Department of Clinical Immunology Fiona Stanley Hospital Murdoch WA Australia; ^20^ School of Population and Global Health, the University of Western Australia Perth WA Australia; ^21^ Northern Clinical School, Sydney Medical School, Faculty of Medicine and Health, The University of Sydney Sydney New South Wales Australia; ^22^ Northern Beaches Clinical School, Faculty of Medicine and Health Sciences, Macquarie University Sydney New South Wales Australia; ^23^ Department of Clinical Immunology and Allergy Northern Beaches Hospital Sydney New South Wales Australia; ^24^ Department of Clinical Immunology and Allergy Incorporating the Jack Jumper Allergy Program, Royal Hobart Hospital Hobart Tasmania Australia; ^25^ College of Health and Medicine, University of Tasmania Hobart Tasmania Australia; ^26^ Centre for Food Allergy Research (CFAR) Parkville Victoria Australia; ^27^ Sydney Medical School, Faculty of Medicine and Health, the University of Sydney Sydney New South Wales Australia; ^28^ Department of Allergy and Immunology Royal Children's Hospital Melbourne Parkville Victoria Australia

**Keywords:** allergic rhinitis, asthma, drug allergy, eczema, food allergy, National health survey, prevalence

## Abstract

**Background:**

The prevalence of allergic diseases across the Australian population, in all regions and age groups, is not well documented. This study aimed to describe the prevalence and distribution of five allergic diseases (allergic rhinitis, asthma, drug allergy, eczema, and food allergy) and examine differences by sociodemographic factors.

**Methods:**

This study used data from the 2022 cross‐sectional Australian National Health Survey. The survey randomly selected a sample of 13,095 households (with 17,093 participants) living in private dwellings in all Australian states and territories. Questionnaires were completed via face‐to‐face interviews. Allergic rhinitis, asthma, drug allergy, eczema, and food allergy were captured as self‐ or parent‐reported long‐term health conditions. Weighted estimates and relative standard errors were extracted and analysed. Prevalence was calculated using population data from the Australian 2021 Census.

**Results:**

The prevalence of self‐reported current allergic rhinitis in Australia was 23.9% (95% CI: 23.1%–24.8%), food allergy was 7.0% (95% CI: 6.5%–7.5%), drug allergy was 5.2% (95% CI: 4.7%–5.6%), eczema was 1.6% (95% CI: 1.3%–1.9%), and diagnosed asthma was 10.8% (95% CI: 10.2%–11.5%). Food allergy and asthma prevalence were similar across childhood and adulthood, whereas the prevalence of allergic rhinitis increased sharply during early adulthood. Eczema was more common in childhood, while drug allergy was more common in later adulthood. Higher rates of allergic rhinitis, food allergy, and eczema were reported among individuals with more advantaged socio‐economic status. In general, allergic diseases were less commonly reported by Indigenous Australians compared to non‐Indigenous Australians, and by individuals born overseas compared to those born in Australia; however, prevalence varied markedly by region of birth, with some regions exhibiting higher reported rates.

**Conclusions:**

We identified a high overall prevalence of allergic diseases in Australia, with variations across populations. The differences across populations may reflect actual prevalence or disparities in disease recognition, reporting, or access to diagnostic services.

## Key Messages

- Self‐reported allergic rhinitis, food allergy, drug allergy, eczema, and diagnosed asthma were 23.9%, 7%, 5.2%, 1.6%, and 10.8%.
- Prevalence of these five allergic diseases varied by age, state, and region of birth.
- Allergic diseases were less commonly reported by Indigenous Australians and individuals born overseas.

## Graphical abstract

Using data from 17,093 participants in the 2022 Australian National Health Survey, this study is the first to report nationwide prevalence estimates for allergic rhinitis (23.9%), food allergy (7.0%), drug allergy (5.2%), eczema (1.6%) and diagnosed asthma (10.8%) across Australia. The report shows how allergy rates vary by age, geographic region and sociodemographic characteristics.

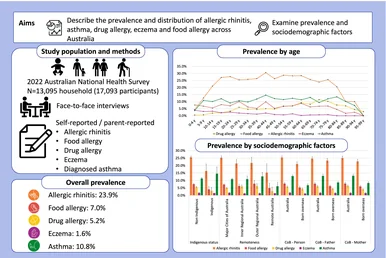

## 1 Introduction

Allergic rhinitis, asthma, drug allergy, eczema and food allergy are chronic conditions that occur when the immune system reacts to usually harmless substances. Allergic diseases negatively impact health, individuals' and families' quality of life, and cause a substantial economic burden [1, 2].

The National Allergy Centre of Excellence (NACE) [3] is Australia's peak allergy research body, established in 2022 to address the significant health and economic burden of allergic diseases in Australia. The centre's purpose is to build critical national research infrastructure and collaboration to drive transformative consumer‐centred allergy care across four streams: drug, food, insect and respiratory allergy. To address recommendations outlined in the Australian Government's 2020 report Walking the allergy tightrope [4], an accurate understanding of the prevalence, burden and socioeconomic disparities in the context of allergic diseases is needed.

The prevalence of allergic diseases across Australia has not been well documented aside from the paediatric population in Victoria's capital city, Melbourne. The HealthNuts study (*n* = 5276) reported a prevalence of food allergy of 11% at age one, reducing to 6% by ages six and ten [5, 6]. In the same cohort, the prevalence of allergic rhinitis increased from 8% at age four to 25% by age ten [5, 6]. Findings from the International Study of Asthma and Allergies in Childhood Phase Three (ISAAC, 2000–2003), also conducted in Melbourne, reported that the prevalence of parent‐ or self‐reported ever having hay fever increased from 19.8% at ages 6–7 to 47.7% at ages 13–14 [7]. From ISAAC (Phase 3), the prevalence of current eczema was 17.1% at ages 6–7, declining to 10.7% at ages 13–14 [8], while ever having asthma increased from 25.5% to 37.3% across the same age groups [9]. In the Longitudinal Study of Australian Children (LSAC), the prevalence of parent‐reported current asthma was 16.0% at ages 6–7 years (measured at 2010–2011) [10].

Data on allergic diseases in adult populations remain limited. A review of studies conducted between 1968 and 2008 reported allergic rhinitis prevalence in Australian adults of 19.2%–47.5% [11]. More recent data found that allergic rhinitis is the second most common chronic condition among young adults (15–24 years), affecting 27% of this age group [12]. Reports of drug allergy prevalence have primarily been restricted to inpatient populations. Reported drug allergy in inpatients ranges from 9% to 18% [13, 14]. The only population data for self‐reported drug allergy comes from South Australia (2017), with a rate of 22% [15]. Overall, there is a lack of consistently collected, population‐level data on allergic diseases across the Australian population.

Previous research has identified disparities in allergy prevalence across population groups. For example, children with Asian heritage have higher rates of nut allergy, eczema, food and insect anaphylaxis compared to other ethnicities [16, 17]. However, existing cohorts have been limited in their ability to explore variations in allergy prevalence across broader sociodemographic domains, particularly in culturally and linguistically diverse communities, due to small sample sizes. There is an urgent need to improve understanding of how allergic diseases vary across sociodemographic characteristics to assist with resource allocation to areas, groups or individuals of greatest need.

The National Health Survey is an Australia‐wide survey [18] that collects information on the prevalence of health conditions and risk factors, along with demographic and socioeconomic characteristics. Notably, this survey includes data on allergic rhinitis, asthma, drug allergy, eczema, and food allergy.

Using the 2022 National Health Survey [18], we examined the prevalence of five allergic diseases across Australia and explored whether they differ by age, state and other sociodemographic characteristics.

## 2 Methods

### 2.1 Study Design and Participants

The National Health Survey collected information on health status, lifestyle factors, and sociodemographic characteristics (e.g., age, sex, country of birth, employment and income). The detailed study design and sampling method can be found on the Australian Bureau of Statistics (ABS) website [18]. In brief, all usual residents in Australia living in private dwellings from urban and rural areas in all states and territories were included in the survey scope. People living in non‐private dwellings (2.9% of the Australian population according to the 2021 Census) [19], such as hotels, hospitals, nursing homes, short‐stay caravan parks and in very remote areas were excluded. Households were randomly selected to participate in the survey. Households completed the first part of the survey collecting basic demographic information about all usual residents of the household via an online form, telephone interview or face‐to‐face interview. One adult (3–18 years) and one child (0–17 years) from selected households were randomly selected to complete individual questionnaires via face‐to‐face interviews.

### 2.2 Data Access

Data from the 2022 National Health Survey (released on 15 December 2023) were accessed in February 2025 through TableBuilder [20] a platform made available to researchers at approved institutions. The extracted data from TableBuilder are ‘count’ data, representing the independent estimates of the in‐scope population, which were weighted based on their probability of being selected in the sample. As only a sample of people in Australia were surveyed, results were converted into estimates for the whole population [18]. Together with the weighted estimates, the corresponding relative standard errors, calculated by the ABS, were also extracted as a measure of sampling error associated with the application of personal and household level survey weights.

### 2.3 Measures

The National Health Survey captured respondents' long‐term health conditions, defined as a medical condition (illness, injury or disability) current at the time of interview, which had lasted, or was expected to last 3–6 months. The classification hierarchy of these health conditions was based on the 10th revision of the International Classification of Diseases. Allergic rhinitis, food allergy, drug allergy, asthma and eczema were captured. Survey participants were defined as having self‐reported current allergic rhinitis, drug allergy, eczema, and food allergy if they answered ‘Yes’ to the following question asked by the interviewer, ‘I would now like to ask you about any other long term health conditions that have lasted or are expected to last, for 6 months or more. Do you have any of these conditions? (Pre‐specified response options included hayfever, food allergy, and drug allergy). Eczema was not listed as a long term health condition in the survey, instead, it could be reported via free‐text entry after selecting ‘none of the above’. The questions on asthma were asked separately. Participants were defined as having self‐reported diagnosed current asthma if they responded ‘Yes’ to the following questions: ‘Have you/Has (first name) ever been told by a doctor or nurse that you have/they have asthma?’ and ‘Have you/Has (first name) had any symptoms (including coughing, wheezing, shortness of breath, chest tightness) of asthma or taken treatment for asthma in the last 12 months?’. Data were self‐reported for respondents aged 3–15 years if consent was sought, and otherwise were parent/guardian reported on the child's behalf if consent was not given or if the child was < 15 years. Data on other allergic diseases (e.g., insect allergy) were not available.

The point estimates and relative standard errors for the number of cases were extracted for allergy outcomes by age group, state, and other sociodemographic characteristics (e.g., Indigenous status, country of birth for survey respondent, country of birth for their parents, household income, index of relative socio‐economic disadvantage, remoteness, and labour force status). Detailed information on the sociodemographic characteristics measures is in Table S1. To minimise the risk of identifying individuals in aggregate statistics, perturbation was used to randomly adjust cell values which involved small random adjustments of the statistics that had a negligible impact on the underlying pattern.

### 2.4 Statistical Analysis

The prevalence of allergy outcomes was calculated using population data from the 2021 Census from the ABS (obtained from TableBuilder) as the denominator and was expressed as a percentage. The age‐, state‐ and demographic‐specific prevalence was calculated using the corresponding counts as the numerator and population data as the denominator. The 95% confidence interval (CI) was calculated using the relative standard error. All analysis was conducted in Microsoft Excel.

### 2.5 Ethics Statement

Minimal risk ethics was approved by the Department of Research Ethics and Governance at The Royal Children's Hospital Melbourne (DERP 4193). No written consent has been obtained from the patients as there is no patient‐identifiable data included.

## 3 Results

### 3.1 Sample Characteristics

The National Health Survey was conducted between January 2022 and April 2023, collecting data from 17,073 individuals across 13,095 households throughout Australia, with a response rate of 56.7%. The response rate for the survey ranged from 45.3% to 63.9% across each state [18]. The sample characteristics of participating households and individuals are summarised in Table 1. Among the participating households, New South Wales (NSW) had the highest proportion of participating households (21%), while Northern Territory (NT) had the lowest (6%), which aligns with the population distribution by state [21]. Among the sampled individuals, 48% were male.

**TABLE 1:** Characteristics of sample households and persons.

Items	*N* (%)
**Household characteristics**	***N* = 13,095**
State and Territory	
NSW	2738 (20.9)
VIC	1804 (13.8)
QLD	1629 (12.4)
SA	1476 (11.3)
WA	1733 (13.2)
TAS	1471 (11.2)
NT	839 (6.4)
ACT	1405 (10.7)
**Persons characteristics**	***N* = 17,073**
Sex	
Male	8252 (48.3)
Age	
0–4	1094 (6.4)
5–9	1042 (6.1)
10–14	1122 (6.6)
15–19	964 (5.7)
20–24	620 (3.6)
25–29	874 (5.1)
30–34	1127 (6.6)
35–39	1208 (7.1)
40–44	1223 (7.2)
45–49	1056 (6.2)
50–54	940 (5.5)
55–59	1008 (5.9)
60–64	1097 (6.4)
65–69	1128 (6.6)
70–74	981 (5.8)
75–79	793 (4.6)
80–84	439 (2.6)
85 years and over	357 (2.1)

*Note:* Sample characteristics by other sociodemographic factors are not available.

Abbreviations: ACT, Australian Capital Territory; NSW, New South Wales; NT, Northern Territory; QLD, Queensland; SA, South Australia; TAS, Tasmania; VIC, Victoria; WA, Western Australia.

### 3.2 Prevalence of Self‐Reported Allergic Rhinitis, Food and Drug Allergy, Eczema and Diagnosed Asthma

The prevalence of self‐reported current allergic rhinitis was 23.9% (95% CI: 23.1%–24.8%), food allergy was 7.0% (95% CI: 6.5%–7.5%), drug allergy was 5.2% (95% CI: 4.7%–5.6%), eczema was 1.6% (1.3%–1.9%), and diagnosed asthma was 10.8% (95% CI: 10.2%–11.5%) (Table 2). Females reported a higher prevalence than males for food and drug allergies and asthma.

**TABLE 2:** Prevalence of self‐reported allergic rhinitis, food allergy, drug allergy, eczema and diagnosed asthma in Australia estimated from the 2022 National Health Survey.

Allergic conditions	Male, % (95% CI)	Female, % (95% CI)	Total, % (95% CI)
Allergic rhinitis	23.2 (22.1–24.3)	24.6 (23.2–25.9)	23.9 (23.1–24.8)
Food allergy	5.8 (5.1–6.4)	8.2 (7.4–9.0)	7.0 (6.5–7.5)
Drug allergy	3.5 (2.9–4.1)	6.9 (6.2–7.5)	5.2 (4.7–5.6)
Eczema	1.4 (1.0–1.8)	1.8 (1.4–2.2)	1.6 (1.3–1.9)
Asthma (diagnosed)	9.4 (8.6–10.2)	12.2 (11.2–13.1)	10.8 (10.2–11.5)

*Note:* 2021 Census population is the denominator for calculating prevalence.

### 3.3 Prevalence by Age Group

The prevalence of food allergy and asthma was stable across age groups (Figure 1 and Table S2). Drug allergy was more common in older adults, while allergic rhinitis rose sharply in early adulthood. Eczema was more common in children and showed a downward trend with age. However, the prevalence estimates of these allergies for some age groups had a high relative standard error greater than 25% and should be interpreted with caution.

**FIGURE 1:**
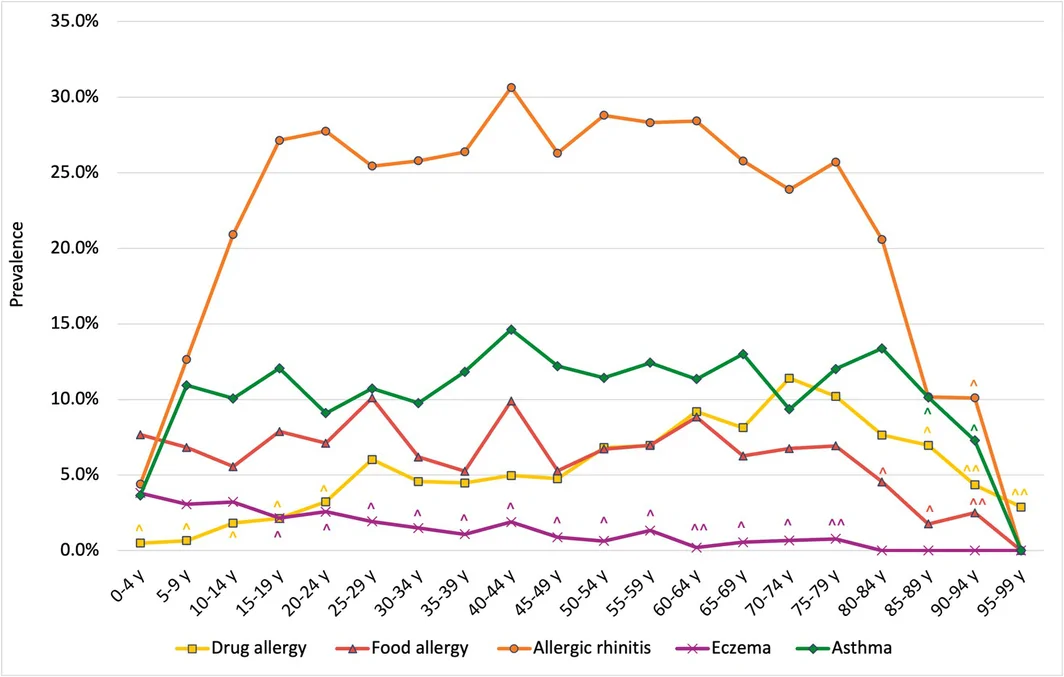
Age‐specific prevalence of self‐reported allergic rhinitis, food allergy, drug allergy, eczema and diagnosed asthma in Australia. ^Estimate has a relative standard error of 25% to 50% and should be used cautiously; ^^Estimate has a relative standard error greater than 50% and is considered too unreliable for general use.

### 3.4 Prevalence by State and Territory

The prevalence of these five allergic diseases varied by regions (Figure 2 and Table S3). The Australian Capital Territory (ACT) and Victoria (VIC) reported a higher prevalence of allergic rhinitis compared to NSW, Queensland (QLD), and NT. Similarly, a higher prevalence of food allergy was observed in ACT and VIC compared to NSW. VIC and Tasmania (TAS) had a higher prevalence of asthma compared to NSW and NT. Western Australia (WA) and TAS had a higher prevalence of drug allergy compared to NSW and NT. TAS had a higher prevalence of eczema compared to NSW and QLD.

**FIGURE 2:**
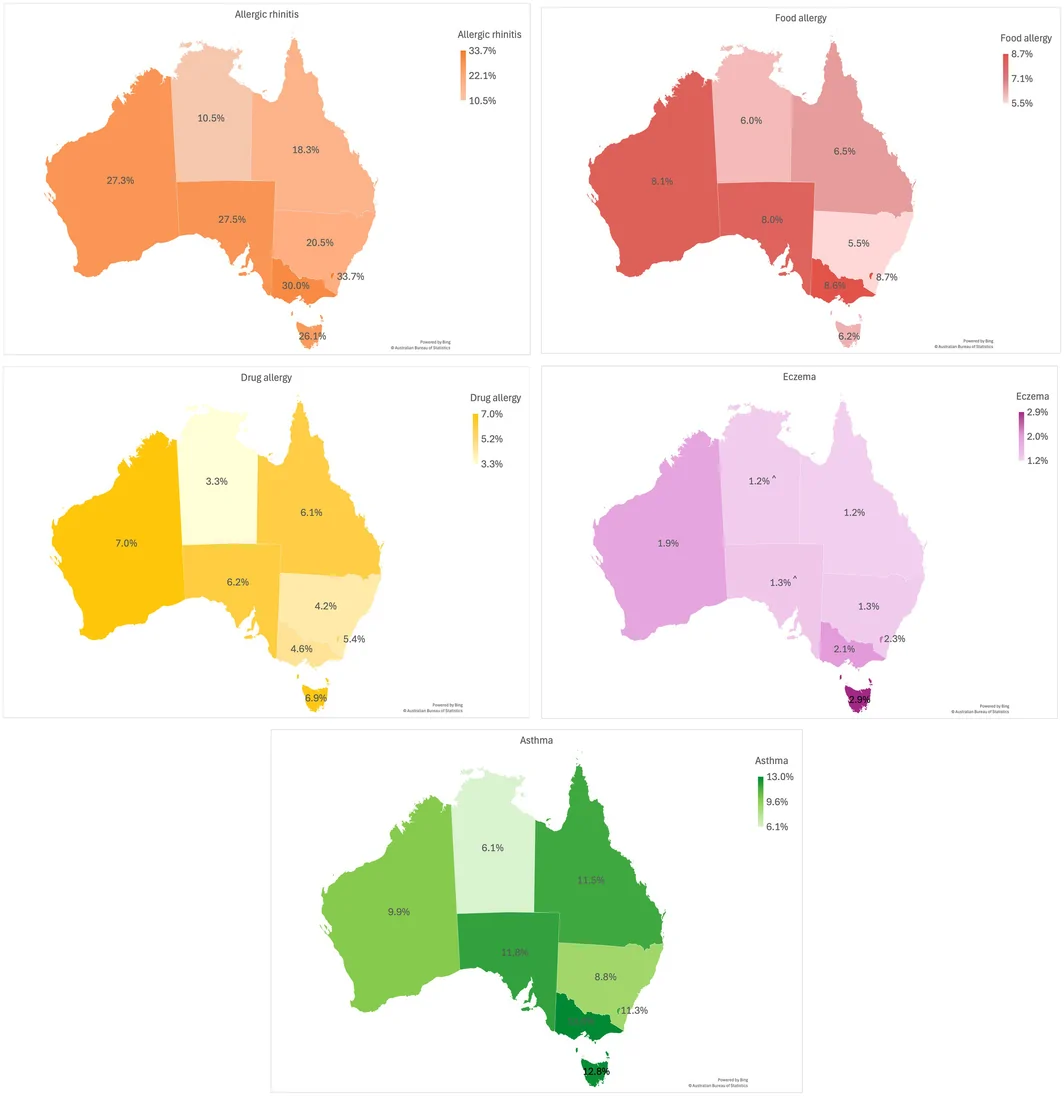
Prevalence of self‐reported allergic rhinitis, food allergy, drug allergy, eczema and diagnosed asthma by state and territory in Australia. ^Estimate has a relative standard error of 25%–50% and should be used cautiously.

### 3.5 Prevalence by Sociodemographic Characteristics

Prevalence also varied by sociodemographic characteristics (Figure 3 and Table S4). Generally, allergic rhinitis, food allergy and eczema showed similar patterns across sociodemographic characteristics. These diseases were more commonly reported by individuals from more advantaged socio‐economic groups, who were employed and had higher household income, although this data should be interpreted with some caution noting the wide confidence intervals in certain subgroups. There was a lack of a clear pattern for asthma and drug allergy based on sociodemographic characteristics.

**FIGURE 3:**
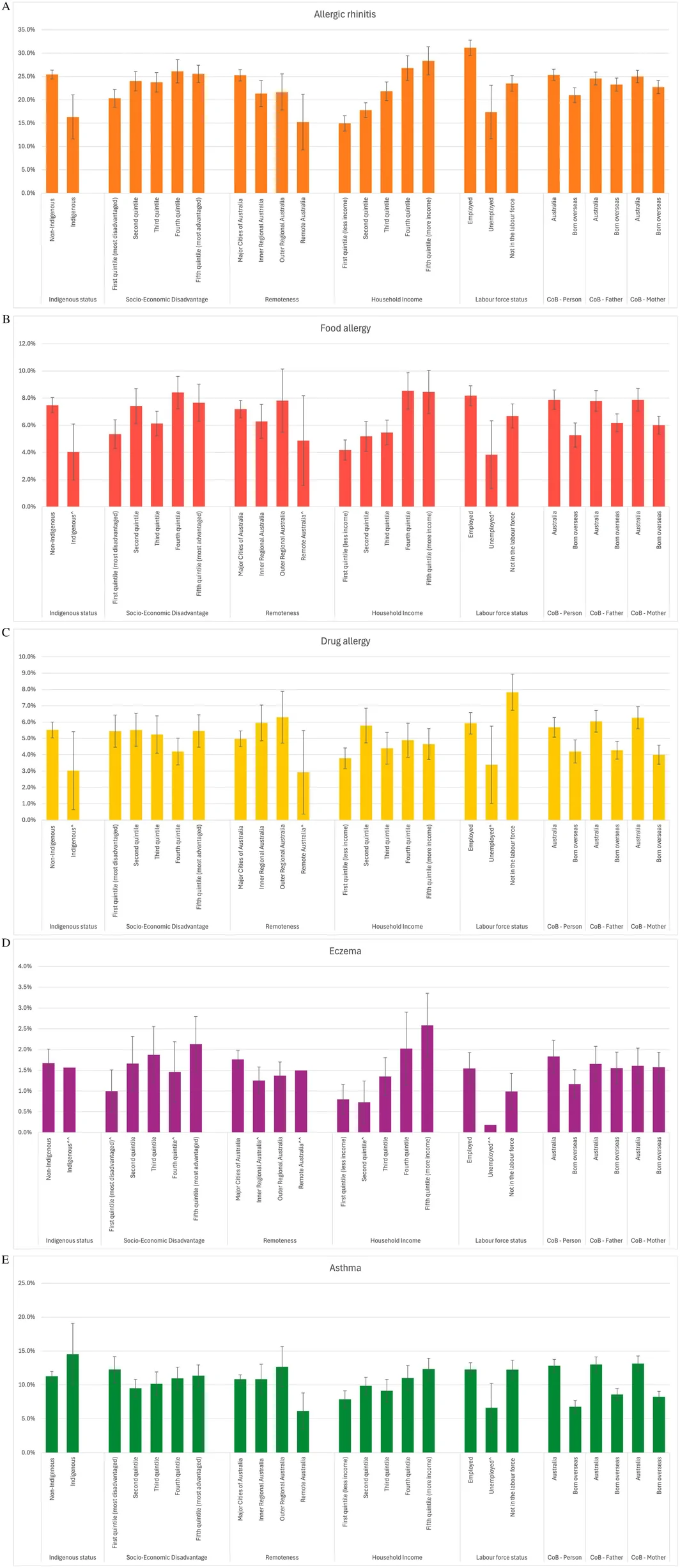
Prevalence of (A) allergic rhinitis, (B) food allergy, (C) frug allergy, (D) eczema, and (E) asthma by sociodemographic characteristics. ^The prevalence estimate for this group has a relative standard error ranging from 25% to 50% and should be interpreted with caution. ^^The prevalence estimate for this group has a relative standard error greater than 50% and should be interpreted with caution. Estimate of 95% CI was not possible because the relative standard error was not provided in the Table Builder output. Labour force status was only available for persons over 15 years old.

Allergic rhinitis and asthma were more commonly reported by those living in major cities (25.3%, 95% CI: 24.0%–26.5% and 10.8%, 95% CI: 10.2%–11.5%) compared to remote Australia (15.2%, 95% CI: 9.3%–21.2% and 6.2%, 95% CI: 3.5%–8.8%).

### 3.6 Prevalence by Indigenous Status

Allergic rhinitis and food allergy were less commonly reported in Indigenous Australians (4.0%, 95% CI: 2.0%–6.1% and 16.3%, 95% CI: 11.6%–21.1%) compared to non‐indigenous Australians (7.5%, 95% CI: 6.9%–8.0% and 25.4%, 95% CI: 24.5%–26.4%). Drug allergy was also less commonly reported among Indigenous Australians, whereas asthma was more commonly reported, noting the wide confidence intervals for this group.

### 3.7 Prevalence by Country of Birth

All five allergic diseases were less common in individuals born overseas compared to those born in Australia (Figure 3). Similarly, food allergy, drug allergy, and asthma were less common in individuals whose parents were born overseas compared to those born in Australia.

Figure 4 and Table S5 present the prevalence of allergic diseases by self and parents' country of birth, which was classified into detailed regions; however, some estimates were imprecise with wide confidence intervals. Individuals born in North‐West Europe had a higher prevalence of reported drug allergy (9.3%, 95% CI: 6.8%–11.9%), compared to individuals born in Australia (5.7%, 95% CI: 5.1%–6.3%) and other overseas regions such as South‐East Asia (3.6%, 95% CI: 1.5%–5.7%). Similar trends were found for individuals whose parents were born in North‐West Europe. For allergic rhinitis, individuals born in or with their parents born in South‐East Asia reported a higher prevalence compared to individuals born in or with their parents born in Australia and other overseas regions such as Europe. There were no clear trends for food allergy, asthma and eczema by these detailed country of birth groups.

**FIGURE 4:**
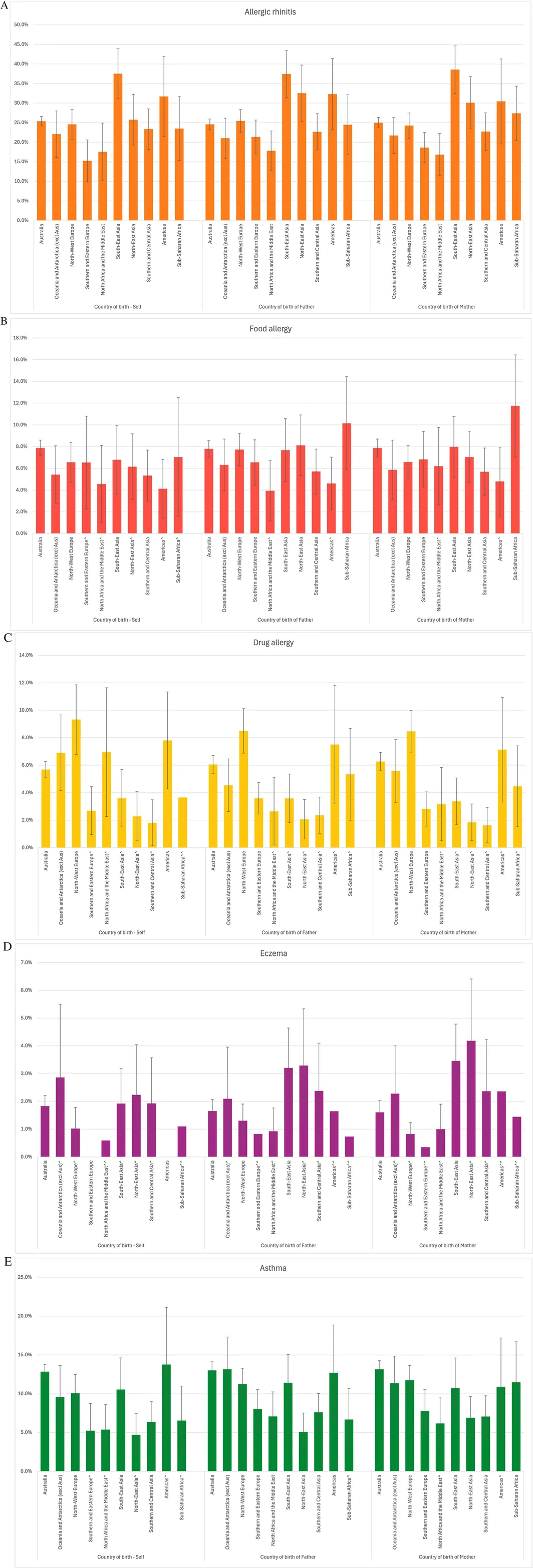
Prevalence of (A) allergic rhinitis, (B) food allergy, (C) drug allergy, (D) eczema, and (E) asthma by self and parents' country of birth. ^The prevalence estimate for this group has a relative standard error ranging from 25% to 50% and should be interpreted with caution. ^^The prevalence estimate for this group has a relative standard error greater than 50% and should be interpreted with caution. Estimate of 95% CI was not possible because the relative standard error was not provided in the Table Builder output.

## 4 Discussion

### 4.1 Principal Findings

Based on data from the 2022 National Health Survey, the prevalence of self‐reported current allergic rhinitis in Australia was 23.9%, food allergy was 7.0%, drug allergy was 5.2%, eczema was 1.6%, and diagnosed asthma was 10.8%, highlighting the extent of the burden of allergic disease across the Australian population. The prevalence of food allergy and asthma was stable with age; however, rates of allergic rhinitis increased sharply in early adulthood. Eczema was more common in children, with a downward trend with increasing age, while drug allergy was more common in later adulthood. Allergic rhinitis, food allergy, and eczema were more commonly reported among more advantaged socio‐economic groups, while there was no clear pattern for drug allergy. Allergic diseases were generally less commonly reported by Indigenous Australians compared to non‐Indigenous Australians, except for asthma. People born in Australia or with Australian‐born parents generally reported a higher prevalence of allergic diseases than those born overseas.

### 4.2 Interpretation in Light of Existing Evidence

This is the first study to report the prevalence of five common allergic diseases—allergic rhinitis, asthma, drug allergy, eczema and food allergy, − across the lifespan in Australia. Consistent with patterns observed in the U.S. [22], the prevalence of food allergy emerged in early childhood and remained relatively stable throughout life. In contrast, drug allergy was more common in late adulthood, aligning with previous findings [23]. This may be attributed to cumulative exposure, higher antibiotic use for infections and medications for age‐related chronic conditions in older adults, which may trigger drug allergy reporting [24, 25] and may uncover previously unrecognised drug allergies. For allergic rhinitis, reported prevalence steadily increased from childhood through early adulthood, consistent with evidence from Asia, Europe and America [26, 27, 28]. We also found a relatively stable prevalence for asthma from childhood to adulthood, whereas previous studies reported a higher prevalence in children [29, 30]. Notably, the prevalence of eczema in our study was lower than that reported in the existing literature, but with a consistent pattern that it's more common in children than adults [31, 32]. The comparatively low prevalence observed may be attributable to the way eczema was reported in the National Health Survey. Self‐reported eczema might underestimate its true prevalence as individuals might miss mild skin symptoms [33]. Moreover, unlike other allergic conditions included in this study, eczema was not available as a pre‐specified response option and could only be reported via free‐text entry. As a result, some respondents may have reported eczema under broader categories such as ‘other allergies’, or not reported their eczema, leading to an underestimate of its true prevalence. Additionally, asthma was defined as self‐reported doctor‐ or nurse‐diagnosed asthma, which differs from the other conditions that were based on self‐report. Therefore, comparisons of prevalence across these conditions should be interpreted with caution, given the differences in measurement methodology.

Allergic rhinitis, food allergy and eczema exhibited similar patterns of variation across sociodemographic characteristics, with higher prevalences reported by individuals from more advantaged socio‐economic groups. This trend was consistent across several indicators, including the area‐level index of Relative Socio‐economic Disadvantage, household income, and labour force status. These findings align with previous research; for example, children of mothers with higher education levels or those living in areas of higher socio‐economic status (SES) were reported to be 35%–64% more likely to have nut allergy or anaphylaxis [16, 34], 27% more likely to have eczema [35] and 21%–59% more likely to have allergic rhinitis [36]. Lifestyle factors associated with SES may contribute to these findings [35, 37, 38, 39].

Such patterns may also reflect differential reporting by SES groups. Underdiagnosis of allergic conditions among disadvantaged and underrepresented minorities has been reported and is often attributed to limited access to specialists, less experience with diagnosis of allergic diseases among primary care providers, and reduced availability of allergy testing [38]. In contrast, individuals from higher SES groups may be more likely to overreport non‐allergic symptoms as allergies [35, 37]. The relationship between SES and allergic diseases is multifaceted, involving factors at the individual, healthcare, community, and policy level [38].

Similarly, the lower prevalence of allergic rhinitis and food allergy among Indigenous Australians compared to non‐Indigenous Australians could reflect a true difference or conversely, underdiagnosis of disease by limited access to allergy care within First Nations communities, due to geographic barriers, cultural considerations and socio‐economic challenges [40]. A recent study using data from 12 public hospitals in Central Queensland (2018–2023) reported significantly higher incidence rates of emergency department presentations related to allergic diseases (e.g., unspecified allergy, atopic dermatitis, anaphylaxis, etc.) among Indigenous Australians compared to non‐Indigenous Australians [41]. The higher rates observed in Indigenous Australians in their study may reflect more severe symptoms, poorer control or limited access to non‐hospital healthcare options and variations in the type of included diseases. Interestingly, although with wide confidence intervals, the higher trend of asthma prevalence in Indigenous population may reflect a true difference or less diagnostic barrier compared to other allergic diseases.

Our study found that individuals born in Australia reported a higher prevalence of allergic diseases compared to those born overseas (classified into one overseas group, see Figure 3), extending previous findings from studies conducted in Victoria to the national level [16]. However, estimates for specific regions should be interpreted with caution due to wide confidence intervals. Individuals themselves or with parents born in north‐west Europe had a higher prevalence of drug allergy compared to those born in Australia or other overseas regions such as South‐East Asia. This is similar to findings from the U.S. that drug allergy was more frequently under‐identified in medical records among patients with limited English proficiency (62.5%) compared to white patients (12%) [42], highlighting how language and healthcare accessibility may contribute to reporting differences. Individuals born in, or with parents born in East Asia reported a higher prevalence of allergic rhinitis compared to those born in Australia or other overseas regions such as Europe, consistent with other reports [43, 44, 45]. However, the aggregated data limited our analysis to define an individual's ethnicity based on their own and parents' country of birth, which further limited the comparability with previous studies.

Our study identified geographical variation in the prevalence of these allergic diseases within Australia, which may have implications for healthcare planning and resource allocation. While this study was not able to determine the underlying drivers of this geographical variation, several differences in state‐level characteristics may plausibly contribute to the observed pattern, including healthcare resourcing, urban and remote population proportions, lifestyle differences and climate. Given that pollen is a key trigger for seasonal allergic rhinitis, regional differences in grass pollen prevalence and distribution across Australia may help explain the observed variations in allergic rhinitis prevalence [46].

### 4.3 Strengths and Limitations

A key strength of this study is the use of a nationally representative sample, which enabled simultaneous investigation of allergic rhinitis, asthma, drug allergy, eczema and food allergy. The National Health Survey also enabled, for the first time, a national‐level comparison of the prevalence of these conditions across different population subgroups and throughout the lifespan.

However, there are several limitations. First, the reliance on self‐reported or parent‐reported data for four out of five allergy outcomes may lead to either overestimation or underestimation of prevalence. Many previous studies have found that self‐reported or parent‐reported food allergy outcomes are often overestimated, as they may include conditions such as food intolerance and sensitivities (e.g., gluten sensitivity or lactose intolerance) [22]. It is well known that the prevalence of true, specialist‐confirmed drug allergy is significantly lower than the reported rate [47]. Even in specialist clinics, drug allergy is challenging to define and diagnose. Self‐reported drug allergy may also include non‐allergic adverse drug reactions or drug intolerance rather than immune‐mediated allergy. Despite these limitations, understanding the prevalence of self‐reported drug allergy remains informative, even if most self‐reported drug allergy are not attributable to immunologically mediated mechanisms. A higher prevalence of self‐reported drug allergy indicates a greater need for clinical assessment and delabelling to ensure accurate identification of true drug allergy and to avoid unnecessary antibiotic avoidance. There is also considerable potential for misclassification in reports of allergic rhinitis, as 50%–70% of individuals with allergic rhinitis self‐manage without formal diagnosis or care [48, 49]. Additionally, chronic nasal conditions such as non‐allergic rhinitis, nasal obstruction, or sinus issues can be mistakenly labelled as allergic rhinitis, or vice versa [50]. These misclassifications may introduce potential bias due to varying levels of disease recognition and health literacy across different sociodemographic groups. While self‐report is an acknowledged limitation, it is a common and accepted approach in large‐scale epidemiological studies where clinical verification is not feasible. The study's large sample size, national scope, and coverage of multiple allergic diseases provide insights into population prevalence and geographic variation that would not otherwise be available. Second, the aggregated nature of the data that we could access for this study, restricted more granular analyses and limited the ability to generate detailed population profiles. Comparisons between subgroups controlling for confounding were not possible. Some count data also exhibited high relative standard error affecting the reliability of certain estimates. Third, the 2021 population data was used as the denominator when calculating the prevalence, which might overestimate the prevalence as it has not been adjusted for the population growth between 2021 and the survey period. Fourth, while weighting reduces bias arising from differential response on observed characteristics, residual non‐response bias may remain if participation is associated with unobserved factors not captured by the weighting benchmarks. For example, individuals with greater health awareness or those experiencing perceived allergic symptoms may have been more likely to participate and to report these conditions, which may not be fully accounted for through weighting procedures. The survey methodology may have introduced selection bias, as only individuals living in private dwellings were included, potentially limiting generalisability. Finally, we only report on the prevalence of five allergic diseases as other common allergic conditions were not included in the National Health Survey data (e.g., insect allergy).

### 4.4 Implications and Future Directions

To enhance our understanding of the prevalence and distribution of allergic diseases across sociodemographic groups and throughout the lifespan, there is a critical need for objective, clinically validated, population‐level data. As the first national snapshot of allergy prevalence, our findings provide insight into the risk trends of five key allergic diseases—allergic rhinitis, asthma, drug allergy, eczema and food allergy—across age groups, geographic regions, and sociodemographic subgroups. It is important to highlight that the self‐reported nature of the data in this study may contribute to the disparities in allergic disease prevalence observed across sociodemographic subgroups, as these differences may partly reflect variations in disease recognition and access to diagnostic services. To more accurately assess the prevalence of allergic diseases, future research should incorporate clinically validated data, such as doctor‐diagnosed or gold‐standard diagnostic measures or including reaction symptoms, to address potential underestimation or overestimation associated with self‐reported data.

Notably, the exclusion of insect allergy from the National Health Survey is a significant limitation, given that fatalities from insect allergies account for approximately three times the number of deaths as those from food allergies [51]. This highlights the need to include insect allergy in future National Health Survey data collections and other studies to capture the full spectrum of allergic disease burden.

## 5 Conclusions

In summary, based on data from the 2022 Australian National Health Survey, this study is the first to report national‐level prevalence estimates for self‐reported current allergic rhinitis (23.9%), food allergy (7.0%), drug allergy (5.2%), eczema (1.6%) and diagnosed asthma (10.8%). The prevalence varied by age, geographic region and sociodemographic characteristics. These findings highlight the importance of understanding the variability in allergic disease burden across population subgroups to inform the development of targeted, evidence‐based strategies. Tailored interventions are essential to optimise the allocation of healthcare resources and equitable access to allergy care across Australia.

## Author Contributions

Yichao Wang: conceptualisation, methodology, software, formal analysis, investigation, data curation, writing – original draft, writing – review and editing, visualisation. Jennifer J. Koplin: writing – review and editing. Janet M. Davies: writing – review and editing. Constance H. Katelaris: writing – review and editing. Debra J. Palmer: writing – review and editing. Jason A. Trubiano: writing – review and editing. Joy Lee: writing – review and editing. Michaela Lucas: writing – review and editing. Michael O'Sullivan: writing – review and editing. Sandra Vale: writing – review and editing. Sheryl Van Nunen: writing – review and editing. Troy Wanandy: writing – review and editing. Dianne E. Campbell: writing – review and editing. Kirsten P. Perrett: conceptualisation, funding acquisition, methodology, writing – review and editing, supervision. Rachel L. Peters: conceptualisation, methodology, writing – review and editing, supervision.

## Funding

Yichao Wang is supported by an Executive Dean Health Research Fellowship from Deakin University and a Postdoctoral Fellowship from the Australian Government funded National Allergy Centre of Excellence (NACE). Debra J Palmer is supported by a Stan Perron Charitable Foundation Fellowship. Kirsten P. Perrett is supported by a Melbourne Children's Clinician–Scientist Fellowship and an NHMRC fellowship, GNT2008911. Research at the Murdoch Children's Research Institute is supported by the Victorian Government's Operational Infrastructure Support Program. Janet M. Davies current research is supported by ARC (DP240203307), Medical Research Future Fund (MRFF) (2031254), NACE, and NHMRC (2032605).

## Conflicts of Interest

Kirsten P. Perrett has received research grants from Aravax, DBV Technologies, Novartis and Siolta, and consultant fees from Aravax, Novartis and RAPT Therapeutics, paid to her institution, outside the submitted work; Janet M. Davies’ organisation has received grants from Bayer Healthcare LLC USA for investigator‐initiated contracted research; outside of the submitted work, Joy Lee has received speaker fees from Sanofi, GSK, Inside Practice, United Clinical, National Asthma Council and AstraZeneca, travel grants from GSK, Sanofi and AstraZeneca, and has served on an advisory board for AstraZeneca; Michaela Lucas is the Co‐Chair for the NACE Drug Allergy Stream and current Co‐Chair and Director of the National Allergy Council and has received sitting fees for advisory board membership for CSL Behring, CSL Seqirus and Boehringer Ingelheim; Michael O'Sullivan is a non‐remunerated Director and current President of the Australasian Society of Clinical Immunology and Allergy; Dianne E. Campbell received personal fees from DBV Technologies (employment and shares), personal fees from Westmead Fertility Centre (honorarium), and grants from the National Health and Medical Research Council of Australia and FARE (Food Allergy Research and Education), outside the submitted work during the conduct of the study; all other authors (Yichao Wang, Jennifer J. Koplin, Constance H. Katelaris, Debra J. Palmer, Jason A. Trubiano, Sandra Vale, Sheryl van Nunen, Troy Wanandy, and Rachel L. Peters) declare no conflicts of interest.

## Supporting information


**Table S1:** Measures of the socio‐demographic characteristics.
**Table S2:** Prevalence of self‐reported allergic rhinitis, food allergy, drug allergy, eczema and diagnosed asthma by age group in Australia.
**Table S3:** Prevalence of self‐reported allergic rhinitis, food allergy, drug allergy, eczema and diagnosed asthma by state and territory.
**Table S4:** Prevalence of self‐reported allergic rhinitis, food allergy, drug allergy, eczema and diagnosed asthma by socio‐demographic characteristics.
**Table S5:** Prevalence of self‐reported allergic rhinitis, food allergy, drug allergy, eczema and diagnosed asthma by self and parents' country of birth.

## Data Availability

The data that support the findings of this study are openly available in Australian Bureau of Statistics at https://www.abs.gov.au/statistics/health/health%E2%80%90conditions%E2%80%90and%E2%80%90risks/national%E2%80%90health%E2%80%90survey/latest%E2%80%90release.
