# Supplementary material for: Prevalence and Sociodemographic Variation of Allergic Diseases in Australia: Findings From the Australian National Health Survey
Source: Clin Exp Allergy. 2026 Apr 5;56(10):1191–204. doi: 10.1111/cea.70288 (PMC13630507; doi:10.1111/cea.70288)
Supplement: Supplementary file 1 [file CEA-56-1191-s001.docx]

**Supplementary Table S1.** Measures of the socio-demographic characteristics

| **Socio-demographic characteristics** | **Category** | **Definition** |
| --- | --- | --- |
| Age | 20 groups: 0-4, 5-9, …95-99 years | Five-year age band between 0 and 99 years. |
| States and Territories | 8 categories: New South Wales (NSW), Victoria (VIC), Queensland (QLD), South Australia (SA), Western Australia (WA), Tasmania (TAS), Northern Territory (NT), Australian Capital Territory (ACT) | Australia is broken up into the States and Territories according to the Australian Statistical Geography Standard (ASGS). Eight of the States and Territories were considered in the analysis. Other Territories were not included. |
| Indigenous status | 2 categories: Non-indigenous, Indigenous | Refers to whether the person is of Aboriginal and/or Torres Strait Islander origin, as identified by an adult spokesperson within each household (i.e. not necessarily self-identified). |
| Country of birth | 2 categories for overall analysis: Australia, Overseas;  10 categories for detailed overseas regions classification: Australia, Oceania and Antarctica (excluding Australia), North-west Europe, Southern and Eastern Europe, North Africa and the Middle East, South-east Asia, North-east Asia, Southern and central Asia, Americas, Sub-saharan Africa | Country of birth for each respondent was recorded. To obtain more details on cultural background that may influence health, respondents were asked to identify the country of birth of both their mother and father.  Country of birth for the survey respondents and both of their parents were classified into either Australia or Overseas for the overall analysis, as well as classification according to Standard Australian Classification Countries (SACC) Major group. |
| Household income | 5 categories: First quintile (less income), Second quintile, Third quintile, Fourth quintile, Fifth quintile (more income) | The adult respondent was asked for the applicable combined income total from all sources, before income tax, salary sacrifice or anything else was taken out, and the period that total amount covered (e.g. weekly, fortnightly, etc). Household income was divided into 5 quintiles based on the total current weekly household income from all sources, each comprising 20% of the estimated population. |
| Index of relative socio-economic disadvantage | 5 categories: First quintile (most disadvantaged), Second quintile, Third quintile, Fourth quintile, Fifth quintile (most advantaged) | Socio-Economic Indexes for Areas (SEIFA) is a product developed by the ABS that ranks areas in Australia according to relative socio-economic advantage and disadvantage. The indexes are based on information from the five-yearly Census. SEIFA 2021 was used in this survey. The Index of relative socio-economic disadvantage (IRSD) summarises a range of information about the economic and social conditions of people and households within an area, which is derived from variables such as income, educational attainment, unemployment, and dwellings without motor vehicles. It measures relative disadvantage. A low score indicates relatively greater disadvantage. The index was divided into five quintiles. |
| Remoteness | 4 categories: Major cities of Australia, Inner regional Australia, Outer regional Australia, Remote Australia | The survey utilizes the remoteness areas structure based on the Australian Statistical Geography Standard (ASGS) that divide Australia into five classes of remoteness on the basis of a measure of relative access to services. The five remoteness classes are: Major Cities, Inner Regional, Outer Regional, Remote and Very Remote. People living in very remote areas of Australia were excluded from the survey. |
| Labour force status | 3 categories: Employed, unemployed, not in the labour force | Information on labour force status was obtained for persons aged 15 years and over. Persons were classified as either employed, unemployed or not in the labour force based on criteria relating to whether the person had a job in the week prior to interview, whether those who did not have a job were actively seeking work.  Employed persons were those who reported that in the preceding week they had worked in a job, business or farm, or who had a job but were absent during that week; Unemployed persons were those who were not employed in the reference week and actively looked for work sometime during the previous four weeks and were available to start, or waiting to start within the following four weeks. Persons not in the labour force were those who were not employed or unemployed. |

**Supplementary Table S2.** Prevalence of self-reported allergic rhinitis, food allergy, drug allergy, eczema and diagnosed asthma by age group in Australia (Estimated prevalence % with 95%CI)

| **Age band** | **Allergic rhinitis**  **% (95%CI)** | | **Food allergy**  **% (95%CI)** | | **Drug allergy**  **% (95%CI)** | | **Eczema**  **% (95%CI)** | | **Asthma**  **% (95%CI)** | |
| --- | --- | --- | --- | --- | --- | --- | --- | --- | --- | --- |
| **0-4 y** | 4.4 | (2.4, 6.4%) | 7.7 | (5.1, 10.3%) | 0.5^ | (0.1, 0.9%) | 3.8 | (2.2, 5.4%) | 3.7 | (2.1, 5.3%) |
| **5-9 y** | 12.6 | (9.9, 15.4%) | 6.8 | (4.3, 9.3%) | 0.7^ | (0.1, 1.3%) | 3.1 | (1.6, 4.5%) | 10.9 | (8.2, 13.6%) |
| **10-14 y** | 20.9 | (17.5, 24.4%) | 5.6 | (3.9, 7.2%) | 1.8^ | (0.8, 2.9%) | 3.2 | (1.9, 4.6%) | 10.1 | (7.6, 12.5%) |
| **15-19 y** | 27.1 | (22.4, 31.8%) | 7.9 | (5.6, 10.2%) | 2.1^ | (0.8, 3.5%) | 2.2^ | (0.5, 3.8%) | 12.1 | (9.2, 15.0%) |
| **20-24 y** | 27.7 | (22.8, 32.7%) | 7.1 | (4.3, 9.9%) | 3.2^ | (1.3, 5.2%) | 2.6^ | (0.7, 4.4%) | 9.1 | (5.6, 12.5%) |
| **25-29 y** | 25.4 | (21.5, 29.3%) | 10.1 | (7.7, 12.5%) | 6.0 | (4.0, 8.0%) | 1.9^ | (0.9, 3.0%) | 10.7 | (8.2, 13.2%) |
| **30-34 y** | 25.8 | (22.3, 29.3%) | 6.2 | (4.1, 8.3%) | 4.6 | (2.9, 6.2%) | 1.5^ | (0.2, 2.8%) | 9.8 | (7.6, 12.0%) |
| **35-39 y** | 26.4 | (23.4, 29.4%) | 5.3 | (3.7, 6.8%) | 4.5 | (3.0, 6.0%) | 1.1^ | (0.3, 1.8%) | 11.8 | (9.5, 14.1%) |
| **40-44 y** | 30.6 | (27.2, 34.1%) | 9.9 | (7.7, 12.1%) | 5.0 | (3.4, 6.5%) | 1.9^ | (0.8, 2.9%) | 14.6 | (11.4, 17.9%) |
| **45-49 y** | 26.3 | (22.7, 29.9%) | 5.3 | (3.7, 6.9%) | 4.8 | (3.2, 6.4%) | 0.9^ | (0.3, 1.4%) | 12.2 | (9.4, 15.0%) |
| **50-54 y** | 28.8 | (24.9, 32.7%) | 6.7 | (4.9, 8.6%) | 6.8 | (4.7, 9.0%) | 0.6^ | (0.0, 1.2%) | 11.4 | (8.9, 14.0%) |
| **55-59 y** | 28.3 | (24.1, 32.5%) | 7.0 | (4.8, 9.1%) | 7.0 | (4.9, 9.1%) | 1.3^ | (0.6, 2.1%) | 12.4 | (9.4, 15.5%) |
| **60-64 y** | 28.4 | (24.2, 32.6%) | 8.8 | (6.8, 10.9%) | 9.2 | (6.9, 11.5%) | 0.2^^ | np | 11.4 | (9.0, 13.7%) |
| **65-69 y** | 25.8 | (22.3, 29.3%) | 6.3 | (4.4, 8.1%) | 8.1 | (6.1, 10.2%) | 0.6^ | (0.1, 1.0%) | 13.0 | (10.5, 15.5%) |
| **70-74 y** | 23.9 | (20.2, 27.6%) | 6.8 | (4.5, 9.0%) | 11.4 | (8.7, 14.1%) | 0.7^ | (0.0, 1.3%) | 9.4 | (6.8, 11.9%) |
| **75-79 y** | 25.7 | (21.4, 30.0%) | 6.9 | (4.6, 9.3%) | 10.2 | (7.1, 13.3%) | 0.8^^ | np | 12.0 | (8.0, 16.0%) |
| **80-84 y** | 20.6 | (15.5, 25.7%) | 4.5^ | (2.1, 7.0%) | 7.6 | (4.1, 11.1%) | 0 | - | 13.4 | (8.3, 18.5%) |
| **85-89 y** | 10.2 | (5.6, 14.7%) | 1.8^ | (0.1, 3.4%) | 7.0^ | (1.9, 12.1%) | 0 | - | 10.1^ | (4.7, 15.6%) |
| **90-94 y** | 10.1^ | (3.7, 16.5%) | 2.5^^ | np | 4.3^^ | np | 0 | - | 7.3^ | (1.8, 12.8%) |
| **95-99 y** | 0 | - | 0 | - | 2.9^^ | np | 0 | - | 0 | - |
| **Total** | 23.9 | (23.1, 24.8%) | 7.0 | (6.5, 7.5%) | 5.2 | (4.7, 5.6%) | 1.6 | (1.3, 1.9%) | 10.8 | (10.2, 11.5) |

*Note: CI: confidence interval; ^ Estimate of prevalence has a relative standard error of 25% to 50% and should be used cautiously; ^^ Estimate has a relative standard error greater than 50% and is considered too unreliable for general use; np: estimate of 95%CI was not possible because relative standard error was not provided in the TableBuilder output when greater than 50%.*

**Supplementary Table S3.** Prevalence of self-reported allergic rhinitis, food allergy, drug allergy, eczema and diagnosed asthma by state and territory

| **State** | **Allergic rhinitis**  **% (95%CI)** | | **Food allergy**  **% (95%CI)** | | **Drug allergy**  **% (95%CI)** | | **Eczema**  **% (95%CI)** | | **Asthma**  **% (95%CI)** | |
| --- | --- | --- | --- | --- | --- | --- | --- | --- | --- | --- |
| **New South Wales** | 20.5 | (18.9, 22.1%) | 5.5 | (4.7, 6.3%) | 4.2 | (3.4, 5.0%) | 1.3 | (0.8, 1.7%) | 8.8 | (7.8, 9.9%) |
| **Victoria** | 30.0 | (27.7, 32.4%) | 8.6 | (7.4, 9.9%) | 4.6 | (3.7, 5.5%) | 2.1 | (1.3, 2.9%) | 13 | (11.3, 14.7%) |
| **Queensland** | 18.3 | (16.5, 20.0%) | 6.5 | (5.4, 7.7%) | 6.1 | (4.9, 7.2%) | 1.2 | (0.7, 1.7%) | 11.5 | (9.9, 13.1%) |
| **South Australia** | 27.5 | (25.0, 30.1%) | 8.0 | (6.2, 9.8%) | 6.2 | (4.7, 7.7%) | 1.3^ | (0.7, 2.0%) | 11.8 | (10.3, 13.4%) |
| **Western Australia** | 27.3 | (25.0, 29.7%) | 8.1 | (6.6, 9.6%) | 7.0 | (5.7, 8.4%) | 1.9 | (1.3, 2.6%) | 9.9 | (8.3, 11.4%) |
| **Tasmania** | 26.1 | (23.6, 28.7%) | 6.2 | (4.9, 7.5%) | 6.9 | (5.5, 8.3%) | 2.9 | (1.8, 4.0%) | 12.8 | (10.7, 14.9%) |
| **Northern Territory** | 10.5 | (9.0, 12.0%) | 6.0 | (4.6, 7.4%) | 3.3 | (2.3, 4.3%) | 1.2^ | (0.6, 1.9%) | 6.1 | (4.9, 7.4%) |
| **Australian Capital Territory** | 33.7 | (31.6, 35.8%) | 8.7 | (7.3, 10.1%) | 5.4 | (4.3, 6.4%) | 2.3 | (1.6, 3.0%) | 11.3 | (9.8, 12.9%) |

*Note: CI: confidence interval; ^ Estimate of prevalence has a relative standard error of 25% to 50% and should be used cautiously.*

**Supplementary Table S4.** Prevalence of self-reported allergic rhinitis, food allergy, drug allergy, eczema and diagnosed asthma by socio-demographic characteristics (Estimated prevalence % with 95%CI)

| **Group** | **Category** | **Allergic rhinitis**  **% (95%CI)** | | **Food allergy**  **% (95%CI)** | | **Drug allergy**  **% (95%CI)** | | **Eczema**  **% (95%CI)** | | **Asthma**  **% (95%CI)** | |
| --- | --- | --- | --- | --- | --- | --- | --- | --- | --- | --- | --- |
| **Indigenous status** | Non-Indigenous | 25.4 | (24.5, 26.4%) | 7.5 | (6.9, 8.0%) | 5.5 | (5.0, 6.0%) | 1.7 | (1.3, 2.0%) | 11.3 | (10.6, 12.0%) |
|  | Indigenous | 16.3 | (11.6, 21.1%) | 4.0^ | (2.0, 6.1%) | 3.0^ | (0.6, 5.4%) | 1.6^^ | np | 14.5 | (10.0, 19.1%) |
| **Socio-Economic Disadvantage** | First quintile (most disadvantaged) | 20.3 | (18.4, 22.2%) | 5.3 | (4.3, 6.4%) | 5.4 | (4.5, 6.4%) | 1.0^ | (0.5, 1.5%) | 12.3 | (10.4, 14.2%) |
|  | Second quintile | 24.0 | (22.0, 26.1%) | 7.4 | (6.1, 8.7%) | 5.5 | (4.5, 6.5%) | 1.7 | (1.0, 2.3%) | 9.5 | (8.2, 10.8%) |
|  | Third quintile | 23.8 | (21.7, 25.8%) | 6.1 | (5.2, 7.0%) | 5.2 | (4.1, 6.4%) | 1.9 | (1.2, 2.6%) | 10.2 | (8.4, 11.9%) |
|  | Fourth quintile | 26.1 | (23.6, 28.6%) | 8.4 | (7.2, 9.6%) | 4.2 | (3.4, 5.0%) | 1.5^ | (0.7, 2.2%) | 11 | (9.3, 12.6%) |
|  | Fifth quintile (most advantaged) | 25.6 | (23.7, 27.4%) | 7.7 | (6.3, 9.0%) | 5.5 | (4.5, 6.5%) | 2.1 | (1.5, 2.8%) | 11.4 | (9.8, 13.0%) |
| **Remoteness** | Major Cities of Australia | 25.3 | (24.0, 26.5%) | 7.2 | (6.5, 7.8%) | 5.0 | (4.5, 5.5%) | 1.8 | (1.6, 2.0%) | 10.8 | (10.2, 11.5%) |
|  | Inner Regional Australia | 21.4 | (18.6, 24.1%) | 6.3 | (5.0, 7.5%) | 6.0 | (4.9, 7.0%) | 1.3^ | (0.9, 1.6%) | 10.9 | (8.7, 13.1%) |
|  | Outer Regional Australia | 21.7 | (17.8, 25.5%) | 7.8 | (5.5, 10.1%) | 6.3 | (4.7, 7.9%) | 1.4 | (1.0, 1.7%) | 12.7 | (9.7, 15.7%) |
|  | Remote Australia | 15.2 | (9.3, 21.2%) | 4.9^ | (1.6, 8.2%) | 2.9^ | (0.4, 5.5%) | 1.5^^ | np | 6.2 | (3.5, 8.8%) |
| **Household Income** | First quintile (less income) | 15.0 | (13.3, 16.6%) | 4.2 | (3.4, 4.9%) | 3.8 | (3.2, 4.4%) | 0.8 | (0.4, 1.2%) | 7.9 | (6.6, 9.1%) |
|  | Second quintile | 17.8 | (16.2, 19.4%) | 5.2 | (4.1, 6.3%) | 5.8 | (4.7, 6.8%) | 0.7^ | (0.2, 1.2%) | 9.9 | (8.6, 11.1%) |
|  | Third quintile | 21.8 | (19.9, 23.8%) | 5.5 | (4.6, 6.4%) | 4.4 | (3.4, 5.4%) | 1.4 | (0.9, 1.8%) | 9.1 | (7.5, 10.8%) |
|  | Fourth quintile | 26.8 | (24.2, 29.5%) | 8.5 | (7.2, 9.9%) | 4.9 | (3.8, 5.9%) | 2 | (1.1, 2.9%) | 11 | (9.2, 12.9%) |
|  | Fifth quintile (more income) | 28.4 | (25.4, 31.4%) | 8.5 | (6.9, 10.0%) | 4.7 | (3.7, 5.6%) | 2.6 | (1.8, 3.4%) | 12.4 | (10.8, 13.9%) |
| **Labour force status** | Employed | 31.2 | (29.5, 32.8%) | 8.2 | (7.5, 8.9%) | 5.9 | (5.3, 6.6%) | 1.5 | (1.2, 1.9%) | 12.3 | (11.3, 13.3%) |
|  | Unemployed | 17.4 | (11.6, 23.1%) | 3.8^ | (1.3, 6.3%) | 3.4^ | (1.0, 5.8%) | 0.2^^ | np | 6.6^ | (3.1, 10.2%) |
|  | Not in the labour force | 23.5 | (21.9, 25.2%) | 6.7 | (5.8, 7.6%) | 7.8 | (6.7, 8.9%) | 1 | (0.5, 1.4%) | 12.3 | (10.9, 13.6%) |
| **Country of birth - Self** | Australia | 25.4 | (24.2, 26.6%) | 7.9 | (7.2, 8.6%) | 5.7 | (5.1, 6.3%) | 1.8 | (1.4, 2.2%) | 12.8 | (11.9, 13.8%) |
|  | Born overseas | 21.0 | (19.4, 22.6%) | 5.3 | (4.4, 6.2%) | 4.2 | (3.5, 4.9%) | 1.2 | (0.8, 1.5%) | 6.8 | (5.8, 7.7%) |
| **Country of birth - Father** | Australia | 24.6 | (23.2, 26.0%) | 7.8 | (7.0, 8.5%) | 6.0 | (5.4, 6.7%) | 1.7 | (1.2, 2.1%) | 13 | (11.9, 14.1%) |
|  | Born overseas | 23.3 | (21.9, 24.7%) | 6.2 | (5.5, 6.8%) | 4.3 | (3.7, 4.8%) | 1.6 | (1.2, 1.9%) | 8.6 | (7.7, 9.5%) |
| **Country of birth - Mother** | Australia | 25.0 | (23.7, 26.3%) | 7.9 | (7.0, 8.7%) | 6.3 | (5.6, 6.9%) | 1.6 | (1.2, 2.0%) | 13.1 | (12.0, 14.3%) |
|  | Born overseas | 22.7 | (21.3, 24.2%) | 6.0 | (5.4, 6.7%) | 4.0 | (3.4, 4.6%) | 1.6 | (1.2, 1.9%) | 8.2 | (7.4, 9.1%) |

*Note: CI: confidence interval; ^ Estimate of prevalence has a relative standard error of 25% to 50% and should be used cautiously; np: estimate of 95%CI was not possible because relative standard error was not provided in the TableBuilder output when greater than 50%.*

**Supplementary Table S5.** Prevalence of self-reported allergic rhinitis, food allergy, drug allergy, eczema and diagnosed asthma by self and parents’ country of birth (Estimated prevalence % with 95%CI)

| **Group** | **Category** | **Allergic rhinitis**  **% (95%CI)** | | **Food allergy**  **% (95%CI)** | | **Drug allergy**  **% (95%CI)** | | **Eczema**  **% (95%CI)** | | **Asthma**  **% (95%CI)** | |
| --- | --- | --- | --- | --- | --- | --- | --- | --- | --- | --- | --- |
| **Country of birth - Self** | Australia | 25.4 | (24.2, 26.6%) | 7.9 | (7.2, 8.6%) | 5.7 | (5.1, 6.3%) | 1.8 | (1.4, 2.2%) | 12.8 | (11.9, 13.8%) |
|  | Oceania and Antarctica (excl Aus) | 22.1 | (16.1, 28.0%) | 5.4 | (2.8, 8.1%) | 6.9 | (4.1, 9.7%) | 2.9^ | (0.2, 5.5%) | 9.6 | (5.5,13.6%) |
|  | North-West Europe | 24.6 | (20.8, 28.4%) | 6.6 | (4.7, 8.4%) | 9.3 | (6.8, 11.9%) | 1.0^ | (0.3, 1.8%) | 10.1 | (7.7, 12.5%) |
|  | Southern and Eastern Europe | 15.3 | (9.9, 20.6%) | 6.5^ | (2.3, 10.8%) | 2.7^ | (0.9, 4.4%) | 0 | (0.0, 0.0%) | 5.2^ | (1.7, 8.7%) |
|  | North Africa and the Middle East | 17.5 | (10.2, 24.9%) | 4.6^ | (1.0, 8.1%) | 6.9^ | (2.3, 11.6%) | 0.6^^ | np | 5.4^ | (2.1, 8.6%) |
|  | South-East Asia | 37.5 | (31.1, 43.9%) | 6.8 | (3.6, 9.9%) | 3.6^ | (1.5, 5.7%) | 1.9^ | (0.7, 3.2%) | 10.5 | (6.5, 14.6%) |
|  | North-East Asia | 25.8 | (19.3, 32.3%) | 6.1^ | (3.1, 9.2%) | 2.3^ | (0.5, 4.1%) | 2.2^ | (0.4, 4.0%) | 4.7^ | (2.0, 7.5%) |
|  | Southern and Central Asia | 23.3 | (18.2, 28.5%) | 5.3 | (3.0, 7.7%) | 1.8^ | (0.1, 3.5%) | 1.9^ | (0.3, 3.6%) | 6.4 | (3.7, 9.0%) |
|  | Americas | 31.7 | (21.5, 41.9%) | 4.1^ | (1.4, 6.8%) | 7.8 | (4.3, 11.3%) | 0 | (0.0, 0.0%) | 13.8^ | (6.4, 21.1%) |
|  | Sub-Saharan Africa | 23.5 | (15.3, 31.7%) | 7.0^ | (1.6, 12.5%) | 3.7^^ | np | 1.1^^ | np | 6.5^ | (2.1, 11.0%) |
| **Country of birth - Father** | Australia | 24.6 | (23.2, 26.0%) | 7.8 | (7.0, 8.5%) | 6.0 | (5.4, 6.7%) | 1.7 | (1.2, 2.1%) | 13 | (11.9, 14.1%) |
|  | Oceania and Antarctica (excl Aus) | 21.0 | (15.9, 26.2%) | 6.3 | (3.9, 8.7%) | 4.5 | (2.6, 6.5%) | 2.1^ | (0.2, 4.0%) | 13.1 | (9.0, 17.3%) |
|  | North-West Europe | 25.4 | (22.5, 28.3%) | 7.7 | (6.2, 9.2%) | 8.5 | (6.9, 10.1%) | 1.3 | (0.7, 1.9%) | 11.2 | (9.2, 13.3%) |
|  | Southern and Eastern Europe | 21.3 | (17.0, 25.7%) | 6.5 | (4.5, 8.6%) | 3.6 | (2.4, 4.7%) | 0.8^^ | np | 8 | (5.5, 10.5%) |
|  | North Africa and the Middle East | 17.8 | (12.7, 22.9%) | 3.9^ | (1.2, 6.7%) | 2.6^ | (0.2, 5.1%) | 0.9^ | (0.1, 1.8%) | 7.1 | (3.9, 10.2%) |
|  | South-East Asia | 37.4 | (31.5, 43.4%) | 7.7 | (4.8, 10.6%) | 3.6^ | (1.8, 5.4%) | 3.2 | (1.8, 4.6%) | 11.4 | (7.8, 15.0%) |
|  | North-East Asia | 32.5 | (25.3, 39.7%) | 8.1 | (5.3, 10.9%) | 2.1^ | (0.6, 3.5%) | 3.3^ | (1.2, 5.3%) | 5.1 | (2.6, 7.5%) |
|  | Southern and Central Asia | 22.7 | (18.0, 27.3%) | 5.7 | (3.6, 7.8%) | 2.4^ | (1.0, 3.7%) | 2.4^ | (0.7, 4.1%) | 7.6 | (5.2, 10.0%) |
|  | Americas | 32.3 | (23.2, 41.4%) | 4.6^ | (2.2, 7.0%) | 7.5^ | (3.2, 11.8%) | 1.6^^ | np | 12.7 | (6.6, 18.8%) |
|  | Sub-Saharan Africa | 24.5 | (16.8, 32.1%) | 10.1 | (5.9, 14.4%) | 5.3^ | (2.0, 8.7%) | 0.7^^ | np | 6.7^ | (2.7, 10.7%) |
| **Country of birth - Mother** | Australia | 25.0 | (23.7, 26.3%) | 7.9 | (7.0, 8.7%) | 6.3 | (5.6, 6.9%) | 1.6 | (1.2, 2.0%) | 13.1 | (12.0, 14.3%) |
|  | Oceania and Antarctica (excl Aus) | 21.7 | (17.1, 26.3%) | 5.9 | (3.1, 8.6%) | 5.6 | (3.3, 7.9%) | 2.3^ | (0.6, 4.0%) | 11.4 | (7.9, 14.8%) |
|  | North-West Europe | 24.3 | (21.0, 27.5%) | 6.6 | (5.1, 8.1%) | 8.5 | (7.0, 10.0%) | 0.8^ | (0.4, 1.2%) | 11.7 | (9.8, 13.6%) |
|  | Southern and Eastern Europe | 18.6 | (14.8, 22.5%) | 6.8 | (4.3, 9.4%) | 2.8 | (1.6, 4.1%) | 0.3^^ | np | 7.8 | (5.0, 10.5%) |
|  | North Africa and the Middle East | 16.8 | (11.4, 22.2%) | 6.2^ | (2.7, 9.7%) | 3.2^ | (0.5, 5.8%) | 1.0^ | (0.1, 1.9%) | 6.2^ | (2.8, 9.6%) |
|  | South-East Asia | 38.5 | (32.5, 44.6%) | 8.0 | (5.2, 10.8%) | 3.4^ | (1.7, 5.1%) | 3.5 | (2.1, 4.8%) | 10.7 | (6.9, 14.6%) |
|  | North-East Asia | 30.1 | (23.4, 36.8%) | 7.0 | (4.7, 9.4%) | 1.8^ | (0.5, 3.2%) | 4.2^ | (2.0, 6.4%) | 6.9 | (4.2, 9.6%) |
|  | Southern and Central Asia | 22.7 | (17.9, 27.5%) | 5.7 | (3.5, 7.9%) | 1.6^ | (0.3, 2.9%) | 2.4^ | (0.5, 4.2%) | 7.1 | (4.4, 9.7%) |
|  | Americas | 30.4 | (19.6, 41.3%) | 4.8^ | (1.6, 7.9%) | 7.1^ | (3.3, 10.9%) | 2.4^^ | np | 10.9^ | (4.6, 17.2%) |
|  | Sub-Saharan Africa | 27.4 | (20.5, 34.3%) | 11.7 | (7.0, 16.4%) | 4.5^ | (1.5, 7.4%) | 1.4^^ | np | 11.5 | (6.3, 16.7%) |

*Note: CI: confidence interval; ^ Estimate of prevalence has a relative standard error of 25% to 50% and should be used cautiously; ^^ Estimate has a relative standard error greater than 50% and is considered too unreliable for general use; np: estimate of 95%CI was not possible because relative standard error was not provided in the TableBuilder output when greater than 50%.*
